# Transactions of the Provincial Medical and Surgical Association, Vol. 1

**Published:** 1833-10-01

**Authors:** 


### THE TRANSACTIONS OF THE PROVINCIAL MEDICAL AND SURGICAL ASSOCIATION. Volume I. Octavo, pp. 442. London, 1833.

IN our last Number, we reviewed some of the papers contained in this volume. In the present Number we shall complete the notice, and put our readers in possession of the spirit or substance of such as we think adapted for our pages. The work contains twenty-one articles; some more immediately practical—some less so. On the whole, the undertaking may be regarded as highly creditable to our provincial brethren, being bold in conception, and not deficient in the execution. If we might venture to offer a hint, it would be, to compress—to present next time a less bulky volume, and to admit more sparingly papers of a general character. Writers and editors of the present day should remember that there are great demands on the time of the reading public, and that the perusal of the daily papers and weekly medical journals alone is sufficient occupation for a man of business. He cannot afford time to read essays not immediately practical and to the point; and if many such are presented in a book, he will probably throw it down with a feeling akin to disappointment or ennui. We are not defending this, we merely state what we fear is a fact, and, as caterers for the public must please the public, we leave those concerned to act as they think fit. We do not wish to sin against our own precepts, and shall, therefore, pass without further preface to the matter before us.

### I. Theory of the Frontal Sinus.

[Art. III. of Transactions.]

This is an article of eight pages, from the pen of our able friend, Dr. Milligan. He deals a severe slap on the phrenologists, with whom, he says, "it is impossible to be serious, without becoming at the same time ridiculous." They may say to the Doctor with Davus—bona verba, quæso.

We shall not take up the cudgels, but proceed to the pith of the paper, which is found in the following quotation, and which cannot well be abridged without injustice to the author's notions.

"Considered in relation to the brain, the membranes, the inner table, and the diploë itself, the outer table presents no other definite organization beyond that of an irregular envelope, which is in some places as thin as a wafer, in others thicker than all the rest of the cranium. But, if we view it from without, we find that every particle of its surface is adapted to some purpose which it has to answer in combination with the soft parts with which it is in contact. Many processes are levers for the muscles; others are merely scabrous surfaces for their insertion; others are condyles for joints; others, organs of hearing; others, organs of fixation; others, of protection; and all this in direct reference to the organs in contact, but without the least relation, that can be discovered, to the encephalon. Hence we are forced to conclude that its projections solely originate under the influence, and for the completion of, functions that are all external to the cranium; and the same thing must necessarily be inferred of the external table, which is merely their substratum. These facts being established from observation, it is very easy to see how the frontal sinus, and other contemporary phenomena, take place. The evolution of the frontal sinus does not commence till the seventh year. At this time the bones of the face are still small and childish, the jaws occupied by the first set of teeth, and no acervus cerebri has yet been formed in the pineal gland. But the Wenzels inform us, from many observations, that, at this age of seven years, the brain has arrived at its full magnitude; and by inspecting their tables, copied in Milligan's Majendie, and taking the cube roots of the respective weights at seven, and the age of majority, it will be found that this averment comes very near the truth, and has only been disputed by those who did not understand how to make use of these tables. Now it is a law in the development of the system, that when any part has arrived at its full magnitude, there occurs a sensible diminution in the circulation of that part, which I have elsewhere named *stagnation, re-stagnation,* and *stasis,* but for which it is not easy to find a proper term. When this state occurs, less blood will necessarily pass into the internal carotid, and so much more will consequently be sent to the face and outer parts of the head by the external carotid, the other branch of that trunk from which they arise at the same instant, namely, the common carotid artery. Hence the infantile and puerile brightness of eye now gradually diminishes in splendour, because it is henceforth supplied by the waning powers of the internal carotid, while the complexion of the cheek, the volume of the face, the evolution of the teeth, the eyebrows, and the still small processes of the outer table of the skull, all sufficiently evince that the energy of the circulation has now found a new direction in the branches of the external carotid. All the parts to which this vessel carries blood, are, from henceforth, developed with nearly as much rapidity as the brain had been in the first septennial period of life. But among the bones hereby so rapidly developed, the nasal bones, the spongy portion of the ethmoidal bone, the external table of the os frontis and the ossa malæ, have their full share. But as the growth of all these bones, except the os frontis, is *forwards,* and as this bone always maintains an exact harmonic continuity with the other bones of the transverse suture, it is necessarily brought forward along with these bones. It cannot carry the diploë along with it, which substance not being so well nourished, will tend to become absorbed. The same effect will be greatly promoted from another cause. The schneiderian membrane growing at the same time with great rapidity, advances through the infundibular passages of the ethmoid bone, is now in immediate contact with the decaying fibres of the diploë connecting the two tables of the frontal bone, which may not yet be absorbed, and passing through amongst these, whilst the pulsation of its vessels causes them to be removed, it gradually scoops out for itself a wedge-shaped cavity between the two tables, which we denominate the frontal sinus. This sinus is not completed till twenty-one, or about the time when the bones of the face are fully perfected; but that this is the mode in which it proceeds, may be determined by inspection of the sinus in skulls, at the different ages from its commencement in the seventh year, to the period of its final development. Having given many facts in confirmation of this theory of the origin of the frontal sinus, in my notes to Majendie's Physiology, editions third and fourth, I shall not enlarge more on the matter here, but conclude by observing that this view of the frontal sinuses does not seem liable to any serious objection. The eminences, therefore, and development of parts, situated over the frontal sinuses, can have no relation to, or congruence with, the parts of the brain within." 67.

We fear that this theory is not so conclusive as Dr. Milligan supposes, and that, when examined, it will prove to amount to very little. To say that the face is developed because more blood is sent to it, is merely to go back one step in the road of causation, and to assert what is almost implied or received as a matter of course. But why is the blood determined to the face? Dr. Milligan will answer—because it ceases to be determined to the brain. Why, then, and *how* does it cease to be determined to the brain? Here we arrive at a full stop, and, seeming to have acquired much by the explanation, we have really learnt no more, than that the development of parts is regulated by the sanguineous supply; but what is the efficient agent in regulating the supply, we know no more than before; and this is on the admission of Dr. Milligan's premises.

But this is only the initiative in his theory. The determination being to the face, it grows, and its bones grow. Those in immediate relation to the os frontis advancing, the outer table of the frontal bone, being attached to them, must advance also, whilst the inner table, being in relation with the brain, does not advance, and hence the vacuity between them—hence the frontal sinus. If, however, it were merely this comparatively accidental circumstance that determined the formation of the sinus, why should we have a *sinus* so constantly? Why should there not be a loose cancellous tissue? Dr. Milligan's answer by anticipation is most unsatisfactory. He says that the diploë, not being so well nourished, is therefore absorbed, and that the pulsating schneiderian membrane scoops out for itself a cavity. We fear that this explanation is tainted with that false philosophy, which made one tissue or system in the body a cause of peculiarity of disposition in other systems or tissues; the action of muscles, for instance, the cause of processes and hollows in bones. If the existence of the frontal sinuses were not constant—not productive of utility—and, consequently, not the evident result of design, we might possibly admit explanations, which have for their basis what cannot be considered but as accidental circumstances. When, however, the design is evident, as we conceive that it is in the case of the frontal sinuses, we have no good right to withdraw this instance from connexion with the general mass. We can see no more difficulty in imagining that the Creator designed the frontal sinuses, and implanted in the organism the disposition to their formation, than that he designed the valves in the heart, and affixed to the germ the property of producing them. We know that all this is a refuge for ignorance, but its confession is better than fallacious theory.

Dr. Milligan, we know, will not be offended at our criticism, because if just, he should not be offended, and if erroneous, he can demonstrate the error.

### II. Some Observations on the Peculiarities of Diseases in Infants and Children. By *J. K. Walker*, M. D. Huddersfield.

[Art. V. Trans.]

This paper occupies twenty-six pages. It is written in a discursive style, and is too deficient in method to admit of any thing approaching to an analysis. We will endeavour to select some interesting facts contained in it, and notice here and there an observation of the author's that may seem original or striking.

Dr. Walker commences by observing, that the late population returns exhibit a very considerable decrement in the rate of infant mortality within the last ten years. In some parts of the country this declension, gratifying as our author terms it, melancholy as a Malthusian would consider it, is less obvious. In some of the manufacturing districts, the number of children who have been buried, under one year old, was one-fourth of the whole number of burials in the last ten years. In one report, the number of children who have been buried, under the age of six, is more than half of the whole number. But, as the highest rate of infant mortality has usually been in large towns, so has the diminution of deaths been the greatest there.

Dr. Walker remarks that the subject of infantile diseases has only lately attracted much attention on the part of scientific men. All must agree that it deserves such.

### *Bad Effects from the Cure of Itch.*

We notice this fact, because we have seen some like it, and because we fear that medical practitioners are too indiscriminate in prescribing sulphur ointment in cases of scabies.

"Very lately I saw a child, Mary Calverly, æt. two years, who lay in a comatose state, the pupils dilated and insensible to light; the respiration interrupted by sighs; pulse weak and irregular; bowels costive. On inquiry, I found that the child for several *months had been troubled with an eruption which the mother called the itch,* which had occupied not only the arms and thighs, but other parts of the body. The eruption not giving way to the internal exhibition of alteratives, the external application of an ointment, consisting of the *unguentum sulphuris,* with a little of the *ung. hydrar. nitrat.* added to it, was successful in producing the desired result about three weeks ago. Since that time the child's health has been on the decline, and she has complained of great pains in the bowels and loss of appetite, succeeded by frequent evening exacerbations of fever, and grinding of the teeth during sleep, &c. About ten days ago, she first felt pain in her head, which has since been followed by stupor and convulsions. At the period my attention was drawn to the case, she had been relieved by the repeated application of leeches to the head, and the continued use of calomel, to which I added the friction of the tartar emetic ointment upon the arms and thighs, wishing to re-produce an eruption on those parts. The relief (if relief it might be called) continued but for a day or two, for the stupor increased, the pulse became weaker, and general convulsions followed, terminating in death." 79.

We have seen three or four cases of fatal muco-enteritis following the speedy cure of scabies, by the use of sulphur ointment. Indeed these cases have made such an impression on us that we do not now venture to prescribe this application without extreme attention to diet, and preparation of the patient by mild laxatives. Practitioners should be very cautious in these cases.

*Effects of Diet on Milk.*

Dr. W. observes that though cow's milk readily coagulates on the addition of acids, healthy human milk will not do so. No coagulation is effected by acids, alcohol, rennet, or infusion of the stomach of the fœtus, at least for some days. Nor has human milk the same tendency to run into acescency that cow's milk exhibits. This is the case, says Dr. W., with such mothers only as have generous diet, for experiments have proved that in mammalia the milk is extremely affected by this agency. Thus, if a suckling bitch is made to live for eight days on vegetable aliment alone, its milk will readily undergo spontaneous separation, and be coagulable by the ordinary means. But if the same bitch be fed solely with raw flesh, the quantity of the milk will be considerably diminished, and it will present alkalescent properties. Whether all these circumstances be strictly true we will not pretend to say, but we will take the liberty of mentioning a fact, in some degree corroborative of the latter statement. The writer of this article happened to make a considerable alteration in his diet, and took daily much animal food with porter and wine. His urine, which had previously been naturally acid, became alkaline, and he experienced great lassitude and mental and physical debility. The urine also displayed a great disposition to throw down the phosphates. He was cured, or at least very greatly relieved by taking the nitric acid, resorting to a more vegetable diet, and limiting himself to one or two glasses of wine daily. Now this is opposed to some notions on diseases of the urinary organs, that have lately attracted much consideration. It has been, and is believed, that a poor diet generally induces an alkaline condition of the urine, while a luxurious diet tends to render it acid. It is obvious that this must be received cum grano salis.

Our author occupies a good many pages to shew that scrophula prevails much in children, and attacks many organs. We see nothing new in this portion of the paper, and the manner is somewhat desultory.

Hydrocephalus is glanced at, and the connexion between the brain and the chylopoïetic viscera dwelt on. The only point we deem it necessary to advert to is contained in the following case. It is advanced as an instance of tendency to coma removed by a tonic plan of treatment.

*Case.* "Mary Shaw, æt. two years, an out-patient of the infirmary, who lay for many weeks in a state of coma, from which it was difficult to rouse her.

There was, at the same time, *excessive emaciation of the entire body*, produced partly by a protracted diarrhœa, and previous inadequate nutrition, so that it was considered by all who saw it, as a hopeless case; and yet this child gradually recovered by the occasional use of carbonate of soda and calomel, in powders, with the aid of chalybeate drops; and under this treatment we were able to extricate the little patient from the state of stupor which wore so menacing an aspect. The diarrhœa gradually gave way, and along with an improved state of the digestive functions, the appetite and strength increased, and nothing has since occurred to retard a complete return to health." 98.

*State of the Urine in Children.*

Dr. W. states justly that this has hitherto attracted little investigation. And yet the importance of the inquiry may be estimated from this fact, that in the lower classes the children suffer most from calculous diseases, and in the upper classes the elderly persons. This is thought to depend chiefly on the unwholesome diet of children in the former class.

Dr. Walker remarks, that where the diet of children is unwholesome, and the consequent disorders of their digestive organs neglected, it is common to find a deposition of red sand in the urine. This, however, will often disappear after the exhibition of alkalies, and, in some instances, the urine of a child undergoes a singular change after a continuance of alkaline remedies. It will no longer turn litmus paper red; in other cases, litmus paper (previously reddened by immersion in healthy urine) is turned blue by that under examination. He has seen soda and magnesia given for a length of time without producing any sensible effect on the urine. This secretion has not always an alkaline tendency in emaciated children. Dr. W. has only seen one case of infantile diabetes, such as has been described by Dr. Venables, although he has been on the look out for the affection. The case was that of a boy, æt. 3½ years, who, but a few months before, had slowly recovered from the measles, and was beginning to regain his strength. Without any apparent cause, his appetite failed, his stools were irregular, and the symptoms assumed the form of remittent fever. No worms were seen in his stools, and the urine was, for some time, little more than common. Nor did it appear of a chyliferous character, and the quantity not, perhaps, so excessive as is recorded by Dr. Venables. About five pints were said to have been voided in one day. Many weeks elapsed before the appetite returned, and the stools became natural. He was relieved by the use of the nitric acid, and subsequently by the sulphate of quinine. The local treatment consisted in the frequent application of spir. terebinth. to the lumbar region, and, occasionally, small blisters to the same part.

*Croup and Laryngeal Spasm.*

Dr. Walker approves of the division of croup into the genuine and the spasmodic. He has seen many instances of relief from the repeated exhibition of lavements charged with turpentine, after leeches had been ineffectually applied to the throat. Laryngeal affections appear to our author to be sometimes epidemic. During the various years in which measles have prevailed among the children of his neighbourhood he has frequently remarked that the fatality was very trifling during the early stage, where the eruption was freely developed; but where that was not the case, or where there was a premature retrocession of the eruption, the respiration often assumed a croupy sound, accompanied with a dry cough. And this was sometimes the case, even when the little patient had passed favourably through an attack of the measles. The plan of treatment was varied according to circumstances; in some leeches were found necessary, in others external applications, such as the spir. terebinth. to the neck; and, where there was reason to believe the development of the rash was deficient, to the calves of the legs.

In some cases of that singular laryngeal spasm, described by Dr. Marshall Hall, little organic change was found after death. In some, there was a turgid state of the cerebral vessels and of the lungs, apparently produced by the violence of the spasm and the struggle for breath. Dr. Walker has seen several cases of this affection.

"One of the last examples of this spasmodic affection, which has fallen under my observation, occurred in an infant, whose health, in other respects, did not materially suffer. The attacks of crowing inspiration returned at intervals, sometimes during the night; occasionally they were accompanied by a rigidity of the thumbs and toes, often with convulsions. At all other times the child was playful and lively. From the kind and able care of Mr. Smith, surgeon, of this town, she received considerable relief. The gums were promptly lanced, and the bowels maintained in an open state with the aid of calomel, and the occasional use of glysters medicated with assafœtid. or spir. terebinth. On the supposition that, from the violence of these attacks, some cerebral affection might supervene, leeches were applied to the neck. From a steady perseverance in these remedies, no advantage was reaped beyond a temporary respite from these spasmodic shocks. They again returned, and the gums were again divided, and counter-irritation applied to the nape of the neck. At the time I was first consulted in the case, the child was slightly relieved by a tonic plan of treatment, and by the sulphas quininæ repeated in small doses. But, on reading those cases of Dr. Marshall Hall, where such striking benefits resulted from a change of air, under similar circumstances, I had no hesitation in recommending a trial of the same plan in this case, and though neither the weather, nor the season of the year, was very auspicious, yet, after a removal to Matlock, the child experienced fewer of these convulsive movements, and, in a few weeks, they ceased entirely, and a rapid amendment in health and strength ensued." 106.

Dr. Walker has often seen the same gratifying effects in the children of the poor, after removal from a damp and unwholesome abode to a dry and airy situation. Dr. W. quotes from Dr. Price's essay on Population the following facts. Before the Act of Parliament was passed, which obliged the parish officers of London and Westminster to send their infant poor to be nursed in the country, *not above one in twenty-four of the poor children received into the workhouses, lived to be a year old;* so that, out of two thousand eight hundred, (the average annual number admitted) 2690 died; whereas, since this measure was adopted, only 450, out of the whole number, died, and the greater part of those deaths occcurred during the three weeks that the children were kept in the workhouses. Dr. W. terms this highly gratifying. We are not so sure of that. Medically speaking, it is delightful enough; but, politically, we fear that the 2240 additional paupers, produced annually by the vicious and the wretched of London and Westminster, are no great gain to a country already overstocked. Dr. Walker is no Malthusian, and benevolence prompts him to exclaim, that this extraordinary progress in the preservation of infantile life is "a rich harvest of reward." We would recommend the Doctor to peruse some of Miss Martineau's Illustrations of Political Economy; they may damp his philoprogenitive ardour.

The next paper is on the "Reciprocal Influence of the Mind and Body of Man in Health and Disease," by Dr. Malden. It is interesting, and the subject well handled; but we must pass it unnoticed, and proceed to more rigid facts.

### III. Cases illustrative of Diseases of the Heart. By *Thos. Jeffreys,* M.D. Liverpool.

[Art. VII. Trans.]

This is a sort of clinical confession from a physician who has been in practice twenty-five years, and has been in the habit of taking notes of all cases of importance. Such a man has always something to say that is worth hearing. The object of the cases which he now brings forward is this. The first series are to shew what extensive organic disease may exist in the *Heart* itself, without a fatal termination; the second, what obscure diagnostics we often have, when the most formidable consequences may be expected: and the third, what powerful symptoms of heart-disease may exist which may be purely symptomatic, and consequently from which no danger may either exist, or be expected. And first of the cases of extensive organic disease. Two are related.

### Case 1.—*Palpitation from Adhesion of the Pericardium.*

We may state that, as disease of the heart and great vessels is rather the disease of advanced than of early life, we are led to investigate the causes of its occasional occurrence in the latter. These are found, on a large induction, to be essentially two—acute rheumatism, and violent mental or physical exertions. Now acute rheumatism is an inflammatory affection, and consists of inflammation affecting chiefly the cellular and fibrous tissues. When it affects the heart, it usually affects these tissues still, pericarditis is consequently set up, and, as its sequence, adhesions of the pericardium are left. Thus it is that, after acute rheumatism, we have frequently adherent pericardium, and every good practitioner, remembering this, should make a point of interrogating every patient who presents himself with a cardiac complaint, as to the prior occurrence or otherwise of rheumatic attacks.

The case before us is one of considerable enlargement of the heart, and intimate adhesions between it and the pericardium, connected with acute rheumatism. We have had so many cases of this description published within these last few years, that we do not think we need notice it more in detail.

### Case 2.—*Supposed Organic Disease, not fatal.*

Thos. H. æt. 40, consulted our author, in Feb. 1828, on account of an affection of the heart which had commenced five years previously. He was successively under the care of Mr. Callan, Dr. Renswick, Dr. Vandeburgh, the late Dr. Macartney, several able physicians and surgeons of Dublin, and the medical officers of one of the dispensaries of Liverpool, all of whom pronounced that the disease was organic and irremediable. Bleeding, blistering, diuretics, change of air, salivation, an issue between the shoulders, and two setons were among the means employed.

At the time of his application to our author, the following was his condition.

Palpitation, easily excited, accompanied by a perceptible fulness, extending up to the left clavicle, and much pain, relieved by holding himself quite erect, and leaning to the opposite side—beating of the left side of the head—a train of dyspeptic symptoms, and great nervous irritability, aggravated, no doubt, by apprehension, and a consciousness of his precarious situation.

Dr. Jeffreys urged him not to despair. He ordered fourteen leeches to be applied between the shoulders, and a bitter infusion to be taken in the state of effervescence, with five grains of the extract of hyosciamus in each dose; a powerful stimulating liniment was ordered to be rubbed freely on the affected side night and morning. He experienced considerable relief from these means, and though he continued to suffer from palpitation and beating in the head, yet by constant employment, simple diet, and attention to his bowels, he was able to pursue his business as a shoemaker. On the 22d of March, 1830, he called upon Dr. Jeffreys, after an interval of two years. The pulse was now 80, regular—the palpitation scarcely perceptible—his looks greatly improved—his spirits good. Exertion of body or agitation of mind would still increase the palpitation, attended with the beating in the head, which was always worse in the recumbent posture.

There can be little doubt that, in this case, there was hypertrophy of the heart; but the inference to be drawn from it is practically important—not to abandon the hope of mitigating a disease, and materially relieving its symptoms, because the cause is of an organic nature.

The next case related by Dr. J. is adduced in support of his second position—that formidable and fatal disease of the heart may be accompanied by symptoms of a very obscure description.

CASE 3.—*Obscure Symptoms of Disease of the Heart—fatal Termination.*

Two cases, indeed, are related under this head. The first was that of a young gentleman who ultimately died, but no examination of the body was obtained. He had been thought, in the first instance, to labour under nervous affection only. The second case, having the demonstrative recommendation of a dissection, is more suited to our purposes.

In April, 1830, Dr. J. visited Hugh Jones, æt. 21, a cabinet maker, who laboured under orthopnœa, cough, expectoration of bloody mucus, constant palpitation of the heart, and pain of the right side of the chest. He was drowsy, easily exhausted, greatly emaciated, and his legs were œdematous. His complaint had commenced four years previously with palpitation, although he had been obliged to pursue his employment until within three weeks of his death.

*Dissection.* "The instant the cartilages of the sternum were divided, a profuse effusion of bloody serum, in the cavity of the chest, made its escape; and upon raising the sternum, the right lung was almost wholly covered with a thick secretion of lymph, which gave it the appearance of ulceration, and upon cutting into it, this state of the lung clearly manifested confirmed phthisis pulmonalis.

The heart, however, was the main object of our inquiry, which we found quite free within its pericardium, with rather more of the liquor pericardii than usual. The heart itself was greatly enlarged; the enlargement was found to be greatest of the right ventricle, having the auriculo-ventricular valve greatly contracted, so that, instead of the passage being of the usual size of a shilling, it would scarcely admit of the passing of a sixpence, and not even this unless passed through edge-ways, presenting the appearance of a long narrow slit, rather than that of a circular opening."

The walls of the left ventricle were thickened, and so were the cordæ tendineæ. The right lung was of a dark colour, and full of tubercles and ulcerations.

The Doctor observes that he feels convinced that this and the former case were alike, and that similar morbid changes would have been found in both. As this is mere conjecture, we need say little about it. We cannot, however, admit that the case we have just quoted is an instance of obscure disease of the heart. A tyro might have detected it. The paper is terminated by two cases of symptomatic or sympathetic affection of the heart. We will give them entire.

### Cases 4 and 5. *Symptomatic Palpitation.*

"Sept. 17, 1828.—Mr. W. C. aged 17, complained much, upon the slightest exertion, of a bounding palpitation of the heart, so violent that, when the stethoscope was applied, it seemed as if the instrument was absolutely raised by the pulsations, which, however, are quite regular. This affection had existed for about a year, and succeeded a sharp attack of pneumonia. He had been attended by a most respectable general practitioner in Shropshire, who considered it a clear case of organic disease, which opinion was strengthened by that of another medical gentleman in the same county. He had also been seen by a celebrated physician there, who prescribed for him, but no very active remedial means had been resorted to. I certainly had very great doubts of its being more than a palpitation from nervous irritability, although there was not any very marked derangement of the digestive organs; but there was such an unusual anxiety for his own feelings and health, that he never left town without being amply provided with medicines and nostrums without end. I put him upon a plan of nervous tonics, internally, and tartar-emetic ointment, alternated with blisters, externally, all with only transient relief. A seton was then inserted in the side, and kept open to his great annoyance, for ten weeks, when it was allowed to heal, on account of its not being so efficacious as was wished for. His general health, however, was greatly improved, and every symptom of irritability gone off, with the exception of the palpitation, which, I believe, continued, more or less, until his mind was vigorously called into action by his going to college; although his anxious solicitude for the preservation of his health, and his minute attention to his own feelings, rendered the experiment somewhat hazardous. It succeeded, however, completely, and in the month of August, 1830, I was informed that he was considered free from complaint, and that, although he did not derive much benefit from the seton at the time, his recovery was mainly attributed to it: and I will not deny that it may have done good, as was proved, beyond all doubt, in the case of Hood, above detailed, who repeatedly assured me that, although the setons were almost more than he could bear, he believed they did him more good than any other remedy. I merely mention this to record the effects of setons, although there was no comparison in the symptoms of the two cases.

I have only a few words to add upon a case of severe Palpitation, occurring in a clergyman, of literary and sedentary habits, in this town, who was so alarmed, either from his own apprehensions, or the prognosis of his medical advisers, that he absolutely was almost afraid of moving from place to place alone. After having gone through a long train of medical discipline, he appeared little or no better. Almost one of the last remedies tried was a seton. Being, however, at last diverted from his sedentary habits, by his election to a popular pulpit, his nervous palpitation left him. I do not know that I should have been warranted in alluding to this gentleman's case, had I not been hastily requested to see him, upon his being suddenly seized with syncope, which alarmed himself and friends so much, that they apprehended the very worst consequences. To me, however, it appeared so decidedly a palpitation from nervous irritability, that I really fear I treated it more lightly than was prudent, and certainly had not the credit of being a true prophet, although the result has fully confirmed my opinion, for he is now in good health, and free from disease, real or imaginary." 140.

This, as we have said, is the termination of the paper. We are always delighted at seeing the publication of the experience of old practitioners, and do every thing in our power to promote it. But the experience of one man is much more important and instructive than that of another. If a man in noting facts, or in relating them, is not careful to observe and to report the differences in particulars, his cases become little more than a catalogue. If for instance, we are presented with a loose description of two cases of affection of the heart, one of which turns out to be organic and fatal, and the other is functional and not fatal, we learn little more than that some diseases of the heart are more dangerous than others. Nay, if there is no good discrimination of symptoms, but a bare enumeration of those which are generic and common to each, we rather lose than gain in point of information, for we feel impelled to conclude, that it is impossible to distinguish two opposite maladies. If, on the contrary, the facts are carefully observed, the points of difference appear, and we learn, not only that the cases were dissimilar, but also the respect in which they were so. This is the kind of experience which is of value to all parties.

We have to regret that the cases of Dr. Jeffreys are open, in some degree, to the imputation of insufficiency. The particulars of difference are not adequately noted—the diagnostic symptoms are not clearly recorded. No cases of disease of the heart should be published now without a statement of the results of stethoscopic examinations.

IV. SOME OBSERVATIONS ON THE VALUE OF THE DIFFERENT SIGNS WHICH DISTINGUISH THE SAC IN STRANGULATED HERNIA; WITH SOME PRACTICAL REMARKS ON THE OPERATION, AND CASES IN ILLUSTRATION. By *J. H. James*, Surgeon to the Devon and Exeter Hospital.

[Art. VIII. Trans.]

The object of Mr. James, a surgeon well known to the profession by his work on Inflammation, is to dispel some of the difficulties not unfrequently experienced by surgeons, in distinguishing the hernial sac from the intestine during operations for hernia. Mr. James is inclined to think that this part of the subject has not received sufficient attention. Mr. James commences by observing that, in old herniæ, the number of investing layers of fascia becomes much increased, a fact often noticed and well known. The difficulty, however, is not so much with large scrotal hernia, old or recent, as with small inguinal, and especially with femoral herniæ. Mr. J. observes that, in the latter, we have beneath the fascia superficialis, which varies much in thickness, the fascia propria, which not merely covers the sac, but completely surrounds it, is precisely of its form, has been denominated by Sir Charles Bell the false sac, and is not unfrequently mistaken for the true one. Secondly, beneath this, and externally to the sac, there is often a considerable quantity of cellular and adipose membrane, divisible frequently into layers, increasing, apparently, the number of investments. Thirdly, the sac itself is thin, often so thin as to be quite transparent, and, in many cases, is distinguishable with difficulty from the intestines. Fourthly, we may have the fascia superficialis *adhering* to the fascia propria, so that they will be divided by the same stroke of the knife, apparently reducing the number of investments divided.

Fifthly, we may, in the same manner, have the fascia propria *adhering* to the true hernial sac.

And lastly, we may have the thin hernial sac either actually adhering to the intestine, or in such close contact with it, as to be nearly, if not quite, inseparable.

Mr. James remarks, that it cannot be a matter of surprise that good surgeons have experienced extreme difficulty in this part of the operation in femoral hernia. Another source of obscurity is the hæmorrhage that sometimes occurs from small vessels. Mr. James does not mention what we have always observed to be the greatest cause of difficulty in femoral herniæ—the presence of an enlarged absorbent gland. The cellular membrane around this becomes condensed into a sort of sac, the gland itself is inflamed and reddened, and on more than one occasion we have seen it mistaken for omentum.

Mr. James passes to the distinguishing characters of intestine and sac. He thinks that they may be divided into those determinable by sight alone, and those by sight and touch conjointly. And first of the former.

Among these will present themselves the number, order, and nature of the external investments, of which nothing more need be said. Two of the indications of the *sac* are its blueish tinge and semi-transparency; the latter, however, often fails when inflammation has increased its thickness, and the former affords no distinction from hernia of the great intestine, which greatly resembles the sac in appearance. Neither indication can be relied on when there is much bleeding. Hæmorrhage, too, will obscure another reputed indication—that the sac speedily becomes dry on exposure, while the intestine does not. The polished appearance of the intestine has been much relied on; this often fails: 1st, from the great thinness of the sac; 2dly, when the contents are great intestine, which differs very little in appearance from sac; and, 3dly, where there is much bleeding. The number and circular direction of the vessels on the intestine, as contra-distinguished from their arborescent appearance and smaller number on the sac, are also mentioned; but, if the bleeding does not obscure them, still the distinction between the sac and *great intestine*, from this cause, is very difficult. In opposition to the blueness of the sac, the dark colour of the intestine has been adduced as a criterion. It is a good one. But it is liable to be obscured by bleeding—sometimes the sac is so thin as to transmit the colour of the intestine—and when the protruded part is omentum or great intestine, this criterion will generally fail. So much, says Mr. James, for the evidence depending on sight alone. And next of the combined indications of touch and sight.

The existence of fluid in the sac is an important particular. But there may be little or no fluid in the sac, or none in the part which we open. In cases of adhesion, there is none of course. It is a good plan to pinch up the part before us, and so ascertain whether it is moveable on the part subjacent. If it be so, that is of course satisfactory. But Mr. James thinks it difficult to judge, if we should pinch up the intestine, of the relative thickness of this and the sac, especially as the latter may be thickened, or the former the thin and distended colon. Where there are adhesions, or where the hernia is tense, and there is no fluid, this indication will prove unserviceable. Again, it is said, if you are outside the sac, you will meet with cellular adhesions, more or less strong, between it and the surrounding parts; while, if you are within, you will be able to pass the probe or the finger easily. Now this, though frequently a true criterion, is not invariably so. On the one hand, adhesions of the intestine may prevent the finger or the probe from passing easily, although the sac *has* been opened; and on the other, it sometimes happens that the probe, or even the finger, may be freely passed outside the sac to the ring, and these are the very cases in which the fatal mistake is sometimes made, of dividing the ring without opening the hernial sac. Mr. J. observes that, although the finger may often be passed outside the sac to the ring, yet the feel of the ring, especially in femoral hernia, will differ from that which is perceived when the finger is inside the sac; it does not appear to pass so completely within its cincture, arising from the attachments of the fascia propria around the ring; and, again, if the ring be divided, we have not, in any case, the same decisive sensation of being able to pass the finger into the abdominal cavity, as when the sac has been opened.

Two other points have not, he thinks, been adverted to, as an indication of opening the sac. The first is the possibility of drawing down more of the intestine, which can generally be done when the sac is opened; the second is the size of the tumour. If it be small intestine, it must, if exceeding the dimensions of an ordinary knuckle, present the appearance of a convolution when the sac is opened; if great intestine, it must exhibit its characteristic appearance of bands.

Omentum is generally recognized by its doughy inelastic feel, before any incision is made, and by its irregular shape after the operation is commenced. Before the sac is opened, its colour will frequently influence that of a thin one; afterwards its granulated feel is distinctive of it.

Mr. James concludes by offering a summary of the practical applications of the foregoing remarks. We will try to make this summary more summary still.

1. The usual investments of herniæ should always be kept in mind. It should also be recollected, that it is chiefly in old herniæ that they become more numerous and complicated. The investments are generally more regular and more distinct in inguinal than in femoral hernia.

2. A thin sac has been commonly observed in femoral hernia. It may be frequently expected in recent herniæ of all descriptions; but it is often met with in large and ancient protrusions.

3. Can we form any judgment, prior to the operation, on the existence of adhesions? If the rupture has been reducible, and the strangulation recent, they are not likely to be encountered; but, if the strangulation has been of long duration, if the inflammation be considerable, and the tumour tense, it is likely that they may exist, but that they will be recent and separable; if, however, the hernia has been an irreducible one, there is a strong presumption that they do exist, and that they are firm.

4. If the tumour is very tense and elastic, it is not only pretty certain that it contains intestine, but that this is likely to be in close approximation with the sac. While, if doughy and inelastic, especially if irregular altogether, or in part, it is commonly understood to contain omentum; or, if simply inelastic, that it contains a considerable portion of fluid. Still this fluid may not interpose at the part where we usually open the sac.

5. A large quantity of fluid in the sac may be inferred, from a diagnostic circumstance pointed out by Sir C. Bell—a portion of the tumour can evidently be returned into the abdomen gradually, without noise, and without relief to the symptoms, but it returns again as soon as the pressure is removed.

6. When there is much pain, heat, and redness of the parts, we may expect considerable bleeding, and should, consequently, consider carefully all assisting circumstances.

7. "In the *progress* of the operation, if we find ourselves baffled in our power of accurately observing the various *visible* phenomena by the bleeding, or by the complicated nature of the investments, then we shall do well to avail ourselves of those other indications which have been now mentioned, and I particularly allude to the power of pinching up a fold separate from a subjacent one, clearly felt; to the power of drawing down more intestine, or the reverse; to the power of passing the finger fairly within the ring, or, if it has been divided, into the abdominal cavity; and in any but very small herniæ, to the power of ascertaining that we have before us a convolution, if small intestine presents, or its characteristic band, if large. If we fail in these points, we have great reason to suppose that it is still hernial sac before us, not opened." 155.

8. We may conclude there is sac before us; but it may be united to the intestine, or in such close juxta-position as to endanger a wound. We must then attend to the following considerations. If the case be one of irreducible hernia, which has become strangulated, there are commonly adhesions; but omentum or fluid may be contained. The omentum may be sought for by the doughy feel, or the irregularity of the tumour at some part; this is an eligible point at which to make the opening. If there is no omentum, there is frequently fluid at the lower part of a bubonocele, and, therefore, if any difficulty is experienced in pinching up the sac, it is better to make the opening in the lower part. Lastly, when irreducible herniæ become strangulated, this accident often supervenes on a recent protrusion; if it be so, we have reason to expect adhesions remote from the ring, while they are not likely to exist near it.

Mr. James relates twelve cases in illustration of the preceding observations. We have not space for them here, nor, indeed, are they necessary for the explanation of what has been advanced. Mr. James has executed his task in a very judicious manner.

The remaining papers in the volume before us, adapted for notice, will be found in another part of this Journal.* They consist of a case of hydroce-

---

* Periscope Department.

phalus—of spina bifida—of aneurism of the basilar artery—and of poisoning by cantharides.

The papers which we cannot advert to, farther than by their enumeration, are these:—An Address at the first Meeting of the Association, by Dr. Hastings—on the Objects and Modes of Medical Investigation, by Dr. Barlow, of Bath—a Proposal to establish County Natural History Societies, by Dr. Conolly, of Warwick—Observations on Sleep, by Dr. Scott, of Liverpool—Report of the Out-patients of the Birmingham Infirmary—Report of the State of Disease in the City of Worcester in the year 1832, by Dr. Streeten—and a Biographical Memoir of the late Dr. Thackeray, of Bedford.

The titles of these papers will shew that, however interesting they may be, and some of them are eminently so, they are not exactly adapted for analysis in a practical journal like this. We must, therefore, leave them, with a recommendation to our readers to consult them in the original volume. The papers which we have already noticed, with the two or three which will be adverted to in our Periscope, will put our readers in possession of a great part of the contents of this provincial volume of Transactions. Though not without faults, both of omission and commission, it is highly creditable to provincial medicine, and we hail it as the harbinger of successors, yet more worthy of our intelligent profession and intellectual age.

---

